# Gli3 Regulates Vomeronasal Neurogenesis, Olfactory Ensheathing Cell Formation, and GnRH-1 Neuronal Migration

**DOI:** 10.1523/JNEUROSCI.1977-19.2019

**Published:** 2020-01-08

**Authors:** Ed Zandro M. Taroc, Ankana S. Naik, Jennifer M. Lin, Nicolas B. Peterson, David L. Keefe, Elizabet Genis, Gabriele Fuchs, Ravikumar Balasubramanian, Paolo E. Forni

**Affiliations:** ^1^Department of Biological Sciences; The RNA Institute, and the Center for Neuroscience Research; University at Albany, State University of New York, Albany, New York 12222, and; ^2^Harvard Reproductive Sciences Center and The Reproductive Endocrine Unit of the Department of Medicine, Massachusetts General Hospital, Boston, Massachusetts 02114

**Keywords:** Ascl-1, Gli3, GnRH-1, Kallmann syndrome, olfactory ensheathing cells, vomeronasal sensory neurons

## Abstract

During mammalian development, gonadotropin-releasing-hormone-1 neurons (GnRH-1ns) migrate from the developing vomeronasal organ (VNO) into the brain asserting control of pubertal onset and fertility. Recent data suggest that correct development of the olfactory ensheathing cells (OEC) is imperative for normal GnRH-1 neuronal migration. However, the full ensemble of molecular pathways that regulate OEC development remains to be fully deciphered. Loss-of-function of the transcription factor Gli3 is known to disrupt olfactory development, however, if Gli3 plays a role in GnRH-1 neuronal development is unclear. By analyzing Gli3 extra-toe mutants (Gli3^Xt/Xt^), we found that Gli3 loss-of-function compromises the onset of achaete-scute family bHLH transcription factor 1 (Ascl-1)^+^ vomeronasal progenitors and the formation of OEC in the nasal mucosa. Surprisingly, GnRH-1 neurogenesis was intact in Gli3^Xt/Xt^ mice but they displayed significant defects in GnRH-1 neuronal migration. In contrast, Ascl-1^null^ mutants showed reduced neurogenesis for both vomeronasal and GnRH-1ns but less severe defects in OEC development. These observations suggest that Gli3 is critical for OEC development in the nasal mucosa and subsequent GnRH-1 neuronal migration. However, the nonoverlapping phenotypes between Ascl-1 and Gli3 mutants indicate that Ascl-1, while crucial for GnRH-1 neurogenesis, is not required for normal OEC development. Because Kallmann syndrome (KS) is characterized by abnormal GnRH-1ns migration, we examined whole-exome sequencing data from KS subjects. We identified and validated a *GLI3* loss-of-function variant in a KS individual. These findings provide new insights into GnRH-1 and OECs development and demonstrate that human *GLI3* mutations contribute to KS etiology.

**SIGNIFICANCE STATEMENT** The transcription factor Gli3 is necessary for correct development of the olfactory system. However, if Gli3 plays a role in controlling GnRH-1 neuronal development has not been addressed. We found that Gli3 loss-of-function compromises the onset of Ascl-1^+^ vomeronasal progenitors, formation of olfactory ensheathing cells in the nasal mucosa, and impairs GnRH-1 neuronal migration to the brain. By analyzing Ascl-1^null^ mutants we dissociated the neurogenic defects observed in Gli3 mutants from lack of olfactory ensheathing cells in the nasal mucosa, moreover, we discovered that Ascl-1 is necessary for GnRH-1 ontogeny. Analyzing human whole-exome sequencing data, we identified a *GLI3* loss-of-function variant in a KS individual. Our data suggest that *GLI3* is a candidate gene contributing to KS etiology.

## Introduction

The olfactory placode gives rise to multiple cell types, including migratory/pioneer neurons, terminal nerve cells, gonadotropin releasing hormone-1 (GnRH-1) neurons (GnRH-1ns), olfactory, and vomeronasal sensory neurons (VSNs) (Wray et al., 1989; Miller et al., 2010; Casoni et al., 2016). GnRH-1ns play a central role in controlling the reproductive axis of vertebrates. During embryonic development GnRH-1ns migrate along the terminal nerve (Schwanzel-Fukuda and Pfaff, 1989; Casoni et al., 2016; Taroc et al., 2017) from the vomeronasal organ into the hypothalamus. Once in the brain the GnRH-1ns control gonadotropin release from the pituitary gland. Defects in neurogenesis and/or migration of GnRH-1ns can lead to Kallmann syndrome (KS), a genetic disorder characterized by pubertal failure due to congenital hypogonadotropic hypogonadism (HH) and anosmia (impaired sense of smell), or to normosmic idiopathic hypogonadotropic hypogonadism (nIHH) (Pingault et al., 2013; Forni and Wray, 2015).

In the last three decades, various causal genes for KS have been identified and disruption of these genetic pathways lead to KS through aberrant development of olfactory placode derivatives and/or impaired migration of GnRH-1ns. Notably, recent studies show that the ability of the GnRH-1ns to migrate from the olfactory pit to the brain is also critically dependent on the correct development of a specialized neural crest derived glial cells termed “olfactory ensheathing cells” (Barraud et al., 2013; Pingault et al., 2013). In keeping with this notion, of the known KS genes, two genes, SOX10 and *ANOS1* have been recently identified as key factors controlling OECs development (Hu et al., 2019). Thus, understanding the genetic pathways governing OEC development could provide more mechanistic clues into the basis of aberrant GnRH neuronal migration and therefore, KS.

Gli3, together with Gli1 and Gli2, are key transcription factors involved in the sonic hedgehog (Shh) signaling. In the absence of Shh signaling, Gli3 and Gli2 act as transcriptional repressors. However, in the presence of Shh, Gli1,2 and Gli3 function as transcriptional activators (Sasaki et al., 1999; Niewiadomski et al., 2014). A spontaneous murine model of the *Gli3* gene, Gli3^pdn/pdn^ has previously implicated a potential role for Gli3 in GnRH neuronal migration. While the hypomorphic Gli3^pdn/pdn^ model shows a delayed, but not missing, GnRH-1 neuronal migration (Naruse et al., 1994), the more severe Gli3-null mutants Gli3 extra-toe (Gli3^Xt/Xt^) fail to develop a functional olfactory system (Keino et al., 1994; Balmer and LaMantia, 2004; Besse et al., 2011). However, GnRH1 neuronal development and migration in Gli3^Xt/Xt^ has not been analyzed.

A candidate gene study in humans has revealed missense variants in the *GLI3* gene in HH (Quaynor et al., 2016) but no functional validation has been performed to ascertain the causality of these variants. Furthermore, *GLI3* mutations in humans have been reported in nonsyndromic forms of polydactyly and syndromic forms of polydactyly of which a subset of patients display neonatal hypogonadism (micropenis and undescended testes) (Johnston et al., 2010). Despite these observations, the precise role of Gli3 in GnRH neuronal biology remains to be fully elucidated.

By analyzing *Gli3*^Xt/Xt^ mutants across the key early developmental stages of embryonic olfactory and GnRH neuronal development, we found that *Gli3* loss-of-function compromises the onset of Ascl-1^+^ vomeronasal progenitors and disrupts the formation of OECs in the nasal mucosa. Notably, in Gli3^Xt/Xt^, although GnRH-1 neurogenesis was preserved as in controls, the GnRH-1ns were unable to migrate and populate the hypothalamus. To further dissect the precise roles of Gli3 and Ascl-1, we also analyzed *Ascl-1-null* mutants and found significant reduction for both vomeronasal and GnRH-1ns but less severe defects in OECs' development than in Gli3^Xt/Xt^, suggesting the OEC development is critically dependent on *Gli3* rather than *Ascl-1*. By whole-exome sequencing of human KS patients, we identified and functionally validated the first *GLI3* loss-of-function variant in a KS individual. These findings provide new insights into GnRH-1 and OECs development and demonstrate that human *GLI3* mutations contribute to KS etiology.

## Materials and Methods

### 

#### 

##### Animals.

Gli3^Xt^ (Schimmang et al., 1992) and Ascl-1^tm1.1(Cre/ERT2)Jeo^/J mice (Kim et al., 2011) on C57BL/6J background were purchased from (Jackson Laboratories). Both colonies were maintained on C57BL/6J. The genotypes of Gli3^Xt^ mice were established by PCR analysis using the following primers: Gli3-C3F: GGCCCA AACATCTACCAACACATAG, Gli3-C3R: GTTGGCTGCTGCATGAAGACTGAC; Gli3-XtJ580F: TACCCCAGCAGGAGACTCAGATTAG; Gli3-XtJ580F: AAACCCGTGGCTCAGGACAAG. Ascl-1^CreERT2^ were genotyped following Ascl-1^tm1.1(Cre/ERT2)Jeo^/J protocol available on jax.org website. Amplification products were analyzed by agarose gel electrophoresis. Animals were killed using CO_2_, followed by cervical dislocation. Mutant and wild-type mice of either sex were used, E15.0 frozen Gli1-LacZ embryos (Gli1^tm2Alj^/J) (Bai et al., 2002) were kindly donated to us by Dr. Melinda Larsen. All mouse studies were approved by the University at Albany Institutional Animal Care and Use Committee.

##### Tissue preparation.

Embryos were collected from time-mated dams where the emergence of the copulation plug was taken as E0.5. Collected embryos were immersion-fixed in 3.7% Formaldehyde/PBS at 4°C for 3–5 h. Postnatal animals were perfused with 3.7% Formaldehyde/PBS. All samples were then cryoprotected in 30% sucrose overnight, then frozen in O.C.T (Tissue-TeK) and stored at −80°C. Samples were cryosectioned using CM3050S Leica cryostat and collected on Superfrost plus slides (VWR) at 12–16 μm thickness for immunostainings and 18–25 μm for *in situ* hybridizations (ISH).

##### Immunohistochemistry.

Primary antibodies and dilutions used in this study were as follows: goat-α-Neuropilin-2 (1:3000; R&D Systems), goat-α-Neuropilin-1 (1:400; R&D Systems), mouse-α-ROBO2(1:50; Santa Cruz Biotechnology) chicken-α-peripherin (1:1500, Abcam), rabbit-α-peripherin (1:2000, Millipore), SW rabbit-α-GnRH-1 (1:6000, Susan Wray; NIH), goat-α olfactory marker protein (1:4000; WAKO), mouse-α-GAD67 (1:200; Santa Cruz Biotechnology), goat-α-AP-2ε (1:1000; Santa Cruz Biotechnology), mouse-α-Meis2 (1:500; Santa Cruz Biotechnology), rabbit-α-phospho-Histone-H3 (1:400; Cell Signaling Technology), rat-α-phospho-Histone-H3 (1:500, Abcam), goat-α-Sox10 (1:100; Santa Cruz Biotechnology), goat-α-Collagen IV (1:800, Millipore), goat-α-Gli3 (1:200; R&D Systems), rabbit-α-Hes-1 (1:200; Cell Signaling Technology), and mouse-α-Ascl-1 (1:30; Santa Cruz Biotechnology). Antigen retrieval was performed in a citrate buffer before incubation with chicken-α-peripherin, rabbit-α-phospho-Histone-H3, rat-α-phospho-Histone-H3, goat-α-AP-2ε, mouse-α-Meis2, goat-α-Gli3, rabbit-α-Hes-1, mouse-α-Ascl-1, goat-α-Sox10, mouse-α-ROBO2, and mouse-α-GAD67 antibodies. For immunoperoxidase staining procedures, slides were processed using standard protocols (Forni et al., 2013) and staining was visualized (Vectastain ABC Kit; Vector Labs) using diaminobenzidine (DAB) in a glucose solution containing glucose oxidase to generate hydrogen peroxide; sections were counterstained with methyl green. For immunofluorescence, species-appropriate secondary antibodies were conjugated with Alexa Fluor-488, Alexa Fluor-594, or Alexa Fluor-568 (Invitrogen and Jackson Laboratories) as specified in the legends. Sections were counterstained with 4′,6′-diamidino-2-phenylindole (1:3000; Sigma-Aldrich) and coverslips were mounted with Fluoro Gel (Electron Microscopy Services). Confocal microscopy pictures were taken on a Zeiss LSM 710 microscope. Epifluorescence pictures were taken on a Leica DM4000 B LED fluorescence microscope equipped with a Leica DFC310 FX camera. Images were further analyzed using FIJ/ImageJ software. Each staining was replicated on at least three different animals for each genotype.

##### *In situ* hybridization.

Digoxigenin-labeled cRNA probes were prepared by *in vitro* transcription (DIG RNA labeling kit; Roche Diagnostics) from the following templates:

Semaphorins 3A (Kagoshima and Ito, 2001), Gli3 riboprobe RP_080717_03_E04 (forward primer GCAGAATTATTCCGGTCAGTTC; reverse primer TCAGTCTTTGTGTTTGTGGTCC). *In situ* hybridization was performed as described previously (Lin et al., 2018) and visualized by immunostaining with an alkaline phosphatase conjugated anti-DIG (1:1000), and NBT/BCIP developer solution (Roche Diagnostics). Sections were then counter-immunostained with antibodies against both chicken-α-peripherin, and SW rabbit-α-GnRH-1, as described above for immunofluorescence.

##### X-gal staining.

Sections were rehydrated in PBS and then incubated in a solution of 5 mm potassium ferrocyanide, 5 mm potassium ferricyanide, 2 mm MgCl_2_, 0.1% Tween, and 0.1% 5-bromo-4-chloro-3-indolyl-b-d-galactoside/dimethylformamide at 37°C O.N. After the enzymatic reaction, slides were counterstained with 1% Eosin-Y (Electron Microscopy Services).

##### Cell quantifications.

Quantification of GnRH-1 neuronal distribution in mutant and control animals was performed in the nasal region (VNO, axonal tracks surrounding the olfactory pits), forebrain junction and brain (all the cells that accessed the olfactory bulb and were distributed within the forebrain). The number of cells was calculated for each animal as the average of the counted cells per series multiplied by the number of series cut per animal. Differences at each interval between genotypes were assessed by unpaired *t* test.

Number of vomeronasal apical and basal neurons was counted after AP-2ε and Meis2 immunostaining, as cells/section on an average of 4 sections/embryo. Progenitor cells were counted after immunostaining for phospho-histone H3, Ascl-1, and Hes-1 and were quantified as described above for vomeronasal cells. Sox10^+^ cells were quantified in a 334,500 μm^2^ field area for an average of 4 VNO sections per animal, only Sox10^+^ cells proximal to the VNO were counted. Frequency of Sox10 cells on vomeronasal bundles was calculated by counting the number of Sox10 I.R. cells/μm fiber length. Means ± SEs were calculated on at least three animals per genotype, *t* test was used to assess differences between groups.

##### Densitometric analysis of the median eminence.

Brains of 5-month-old male Gli3^Xt^ WT and heterozygous mice (*n* = 3) were cryosectioned coronally at 20 μm. Three series for each animal were stained for GnRH in DAB. Brightfield images of comparable sections for each series were taken for quantification. Images were processed with color deconvolution and the mean gray value was measured for a standardized ROI to calculate the average optical density for each animal.

##### Site-directed mutagenesis.

The GLI-3 bs-2 plasmid carrying the human GLI3 (Kinzler et al., 1988) was purchased from Addgene. F387F*fs mutation was generated in the vector pCDNA3 hGli3 using Q5 site directed mutagenesis kit (NEB) using primers Gli3 F387Ffs* Fwd CCAACACAGAGGCCTATTCC and Gli3 F387Ffs* Rev AAAGTTGGGGCAGGGTGG following the manufacturer's instruction. Mutation was validated by sequencing before use in experiments.

##### Transfection and luciferase reporter assay.

1 × 10^4^ HeLa cells (ATCC CCL2) were seeded into a 96-well plate 24 h before transfections. 100 ng of either reporter plasmid (8 × 3 GLI-BS Luc or 8 × 3 Mut GLI-BS Luc) and 100 ng of the effector plasmid (hGli3 WT or F387F*fs) and 20 ng of pRL-SV40 were cotransfected using Lipofectamine 3000 (Thermo Fisher). pRL-SV40 coding for *Renilla* luciferase was used as a transfection control and for normalization. Cells were harvested 48 h posttransfection and Firefly and *Renilla* luciferase intensities were measured using Glomax 96 microplate luminometer (Promega) and Dual-Luciferase Reporter Assay System (Promega).

##### Human phenotyping and genotyping: WES analysis of KS individuals for GLI3 loss-of-function mutations.

A total of 632 KS subjects at the Harvard Reproductive Endocrine Sciences Center were examined for *GLI3* loss-of-function mutations in this study. KS was defined by: (1) absent or incomplete puberty by age 18 years; (2) serum testosterone <100 ng/dL in men or estradiol <20 pg/ml in women in the face of low or normal levels of serum gonadotropins; (3) otherwise normal anterior pituitary function; (4) normal serum ferritin concentrations; (5) normal MRI of the hypothalamic-pituitary region (Balasubramanian and Crowley, 2011); and (6) impaired olfaction as determined by the University of Pennsylvania Smell Identification Test (UPSIT) (Lewkowitz-Shpuntoff et al., 2012; Doty, 2007). Clinical charts, biochemical testing (including overnight neuroendocrine testing for LH pulse profiles) and patient questionnaires of KS subjects were reviewed for phenotypic evaluation as described previously (Pitteloud et al., 2002). Whole-exome sequencing (WES) data in the KS cohort was performed on peripheral blood-derived DNA using either Nimblegen SeqCap target enrichment kit (Roche) or a custom Illumina capture kit (ICE) and the detailed variant-calling and annotation used have been described previously (Guo et al., 2018). WES data were then queried for *GLI3* (RefSeq: NM_000168.6) loss-of-function variants: defined as those variants resulting in protein alteration in the canonical *GLI3* transcript as determined by Variant Effect Predictor (VEP) (127), resulting in nonsense, and essential splice site mutations; or (b) indels resulting in frame-shift mutations; AND (b) with ethnicity-specific minor allele frequency (MAF) <0.1% minor allele frequency (MAF) in gnomAD database (http://gnomad.broadinstitute.org) (Karczewski et al., 2019). To ascertain oligogenicity, KS proband(s) with *GLI3* loss-of-function variants were also examined for rare sequence variants (RSVs) in other known KS/nIHH genes [*ANOS1* (NM_000216.4), *CHD7* (NM_017780.4), *FEZF1* (NM_001160264.2), *FGF8* (NM_006119.4), *FGFR1* (NM_023110.2), *GNRH1* (NM_000825.3), *GNRHR* (NM_000406.2), *HS6ST1* (NM_004807.3), *KISS1* (NM_002256.4), *KISS1R* (NM_032551.5), *NR0B1* (NM_000475.4), *NSMF* (NM_001130969.1), *PROK2* (NM_001126128.2), *PROKR2* (NM_144773.3), *SEMA3A* (NM_006080.3), *SMCHD1* (NM_015295.2), *SOX2* (NM_003106.4), *TAC3* (NM_013251.4), *TACR3* (NM_001059.3), and *WDR11* (NM_018117.12)]. RSVs in these other HH genes were defined as single nucleotide variants resulting in protein alteration in canonical transcript as determined by Variant Effect Predictor (VEP) (127) resulting in missense, nonsense, and essential splice site mutations; or (b) indels resulting in frame-shift mutations; AND (b) with ethnicity-specific minor allele frequency (MAF) <1% in GnoMAD. Variants identified from above analysis were then confirmed by Sanger sequencing. This study was approved by the Partners Institutional Review Board at the MGH, and informed consent was obtained from all participants.

##### Statistical analyses.

All statistical analyses were performed using GraphPad Prism7 software. Cell counts were done on serial sections immunostained for GnRH-1 at E13.5 (*n* = 3) and E15.0 (*n* = 4) and visualized under bright field (immunoperoxidase) or epi-fluorescence illumination (20×; Leica DM4000 B LED), according to their anatomical location: (1) nasal region (VNO, axonal tracks surrounding the olfactory pits, forebrain junction); (2) olfactory bulb/fibrocellular mass; and (3) brain (all the cells that accessed the olfactory bulb and were distributed within the forebrain). For each animal, counts were performed on three serial series. The average number of cells from these three series was then multiplied by the total number of series/animal to compute a value for each animal. These were then averaged ± SE among animals of the same age and genotype. Means ± SEs were calculated on at least three animals per genotype. The statistical difference between genotypes and groups were determined using an unpaired Student's *t* test. A gene-based burden testing for human *GLI3* variants was performed between the KS and the gnomAD using a Fisher's-exact test. All data are represented as the mean ± SEM from *n* ≥ 3 mice per genotype/age for each experiment. Values of *p* < 0.05 were considered to be statistically significant.

## Results

### Gli3 is expressed by apical Hes-1^+^ progenitor cells in the VNO

**Figure 1.**
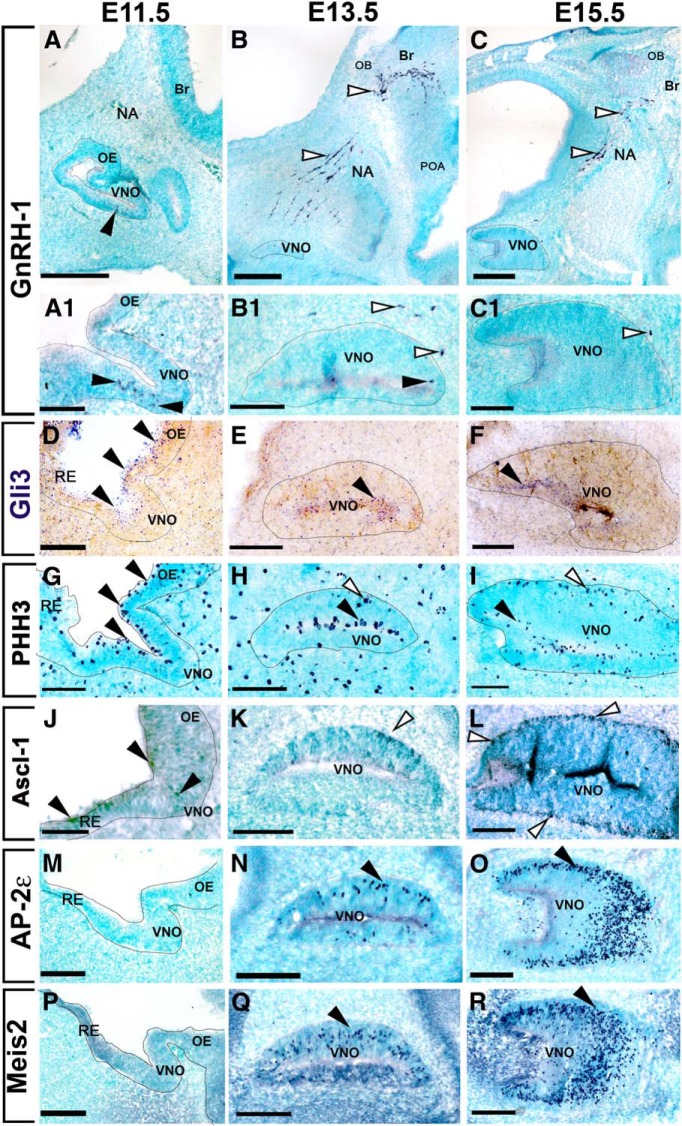
Gli3 is expressed in the developing vomeronasal area. GnRH-1ns can be detected at E11.5 (***A***, ***A1***) in the ventral portion of the developing putative VNO and emerge from the VNO (white arrows) to the nasal area (NA) migrating toward the brain (Br) at E13.5 (***B***, ***B1***) and E15.5 (***C***, ***C1***). ***D***–***F***, Gli3 mRNA expression is detected in cells located in the apical portion of the developing olfactory pit and VNO (black arrows) at all analyzed stages. ***G***–***I***, PHH3 immunoreactivity highlights dividing cells in the apical (black arrow) and basal (white arrows) regions of the developing VNO. ***J***–***L***, Immunostaining against Ascl-1 highlights sparse Ascl-1^+^ cells at E11.5 (***J***), neurogenic Ascl-1^+^ cells were detected in the basal regions of the developing VNO at E13.5 and E15.5. Immunostaining against the basal transcription factor AP-2ε (***M***–***O***) and apical transcription factor Meis2 (***P***–***R***) reveal the lack of detectable vomeronasal neurons at E11.5, while a growing number of neurons were detected between E13.5 and E15.5. Scale bars in ***A***–***C***, 250 μm; ***A1***, ***D***, ***G***, ***J***, ***M***, ***P***, 50 μm; ***B1***, ***C1***, ***E***, ***F***, ***H***, ***I***, ***K***, ***L***, ***N***, ***O***, ***Q***, ***R***, 100 μm.

Because GnRH-1ns originate in the region of the nasal pit that develops into the vomeronasal organ, we analyzed Gli3 expression throughout GnRH-1ns and VSN development. GnRH-1ns arise between E10.5 and E11.5 and are immunodetectable before vomeronasal neuron formation (Fig. 1*A*,*A1*,*M*,*P*) (Forni et al., 2011a, 2013; Forni and Wray, 2015; Taroc et al., 2017). By E13.5–E15.5, we observed that the developing VNO was populated by Tfap2e (AP-2ε)^+^ basal VSNs and Meis2^+^ apical VSNs (Fig. 1*N*,*Q*). However, most GnRH-1ns migrated out of the vomeronasal area by E13.5 (Fig. 1*B*,*B1*,*C*,*C1*).

**Figure 2.**
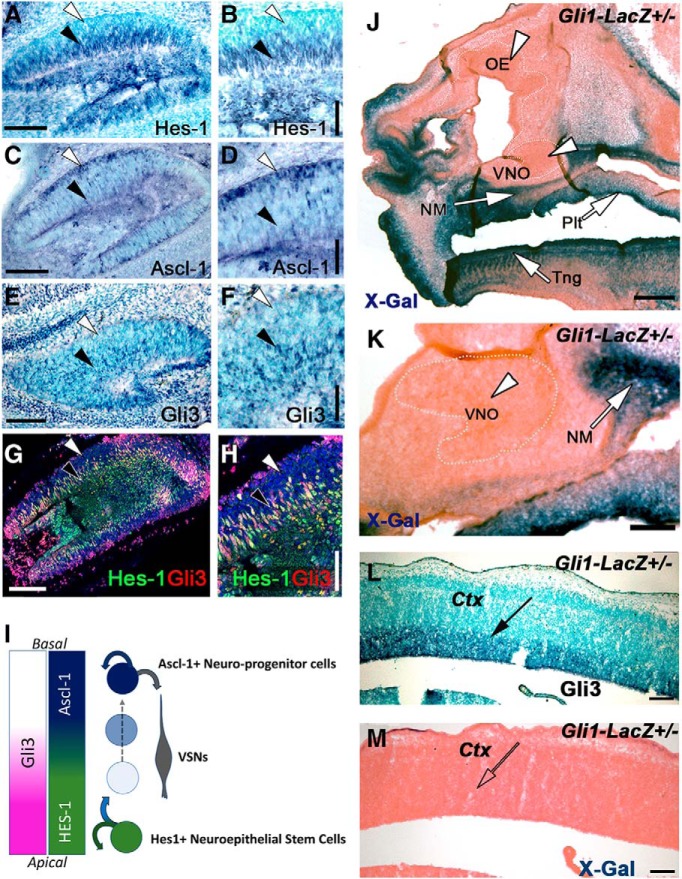
Gli3 is expressed in Hes-1^+^, but not in Ascl-1^+^, progenitors. ***A***, ***B***, E15.5 immunostaining against Hes-1 reveals Hes-1 expression in the cells located in the apical domains of the developing VNO (Black arrow). ***C***, ***D***, Immunostaining against Ascl-1 reveals expression of the neurogenic factor in cells in the basal territories of the developing VNO (White arrow), only background levels were found in the basal regions (Black arrow). ***E***, ***F***, Gli3 immunoreactivity was found in cells in the apical territories of the developing VNO (Black arrow). ***G***, ***H***, Double immunofluorescence against Gli3 and Hes-1 reveals strong Gli3 expression in the Hes-1^+^ cells (Black arrow) and lack of immunodetectable Gli3 in the most basal levels (white arrow). ***I***, Proposed model with Gli3 expression in proliferative Hes-1^+^ cells and in daughter cells that exit the proliferative program and enter the Ascl-1^+^ controlled proneurogenic program in the basal regions of the developing VNO. ***J***, X-Gal reaction on E15.5 sections of Gli1-LacZ Knock-in mouse shows active Shh signaling (Gli1 expression, blue, white arrows) in the palate (plt), nasal mesenchyme (NM), tongue (tng) but not in the olfactory epithelium (OE) nor in VNO (white arrowheads). ***K***, magnification of the VNO showing lack of Gli1 expression. ***L***, ***M***, Serial sections; (***L***) Immunostaining against Gli3 shows strong Gli3 expression in the ventricular zone of the developing brain cortex (arrow), (***M***) X-Gal reaction showing lack of Gli1 expression in the ventricular zone. Scale bars: ***A***, ***C***, ***E***, ***G***, ***K***, ***L***, ***M***, 100 μm; ***B***, ***D***, ***F***, ***H***, ***J***, 50 μm.

We then performed *in situ* hybridization and immunohistochemistry (Data not shown) against Gli3 at embryonic stages E11.5, E13.5, and E15.5 (Fig. 1*D–F*). These experiments revealed that Gli3 mRNA and protein mostly localize in the apical regions of the developing vomeronasal epithelia. The proliferative apical cells in the developing olfactory pit (Cuschieri and Bannister, 1975a) localize Hes-1^+^ stem cell/progenitors that give rise, over time, to apical Ascl-1 neurogenic progenitors (Cau et al., 2000). By performing anti phospho-histone-H3 (PHH3) immunostaining, we only detected mitotic cells in the apical region of the VNO at E11.5. However, at E13.5 and E15.5, we detected mitotic cells along the margins of both the apical and basal regions (Cuschieri and Bannister, 1975b; Cau et al., 2000) (Fig. 1*G–I*). Immunostaining for Hes-1 and Ascl-1 in the developing VNO (Fig. 2*A–D*) showed a progressive enrichment of immunodetectable Ascl-1^+^ cells over time (Fig. 1*J–L*). We confirmed opposite expression patterns for Hes-1 and Ascl-1 at E15.5, as Hes-1 localized predominantly in the apical portions and Ascl-1 localized in the basal portions of the developing VNO (Fig. 2*A–D*). We confirmed Gli3 immunoreactivity in Hes-1^+^ cells and sparse immunoreactivity in the basal regions of the VNO (Fig. 2*G*,*H*).

Following Hedgehog stimulation Gli3, as a transcriptional activator (Gli3a), induces the expression of Gli1 (which is the amplifying activator downstream Shh) (Lee et al., 1997). In the absence of Shh, Gli3 repressor (Gli3R) blocks Gli1 expression (Lee et al., 1997; Dai et al., 1999; Sasaki et al., 1999; Hu et al., 2006). Previous data have suggested that Gli3 acts as transcriptional repressor in the radial glia of the developing brain cortex (Hasenpusch-Theil et al., 2018). To understand if, in the developing VNO, Gli3 acts as an activator or as a repressor we exploited a Gli1 knock-in LacZ mouse line (Bai et al., 2002) as a Hedgehog signaling reporter (Fig. 2*J*,*K*). X-Gal enzymatic reaction revealed Gli1 expression, which is indicative of active Hedgehog signaling (Fig. 2*J*,*K*), in the nasal mesenchyme (NM), cartilage and bones as well as in the oral palate and in the tongue (Sagai et al., 2017). However, even though Gli3 was detected both in the developing brain cortex (Fig. 2*L*) (Hasenpusch-Theil et al., 2018) and VNO (Fig. 2*E–H*), we did not detect Gli1 expression in these areas (Fig. 2*J–M*). Similar results were obtained using anti Gli1 immunostaining (data not shown). Lack of active Hedgehog signaling in the developing OE and VNO, suggests that, in these regions Gli3 acts as a transcriptional repressor.

### Gli3 loss-of-function affects VSN neurogenesis but retains VSN specification and differentiation

**Figure 3.**
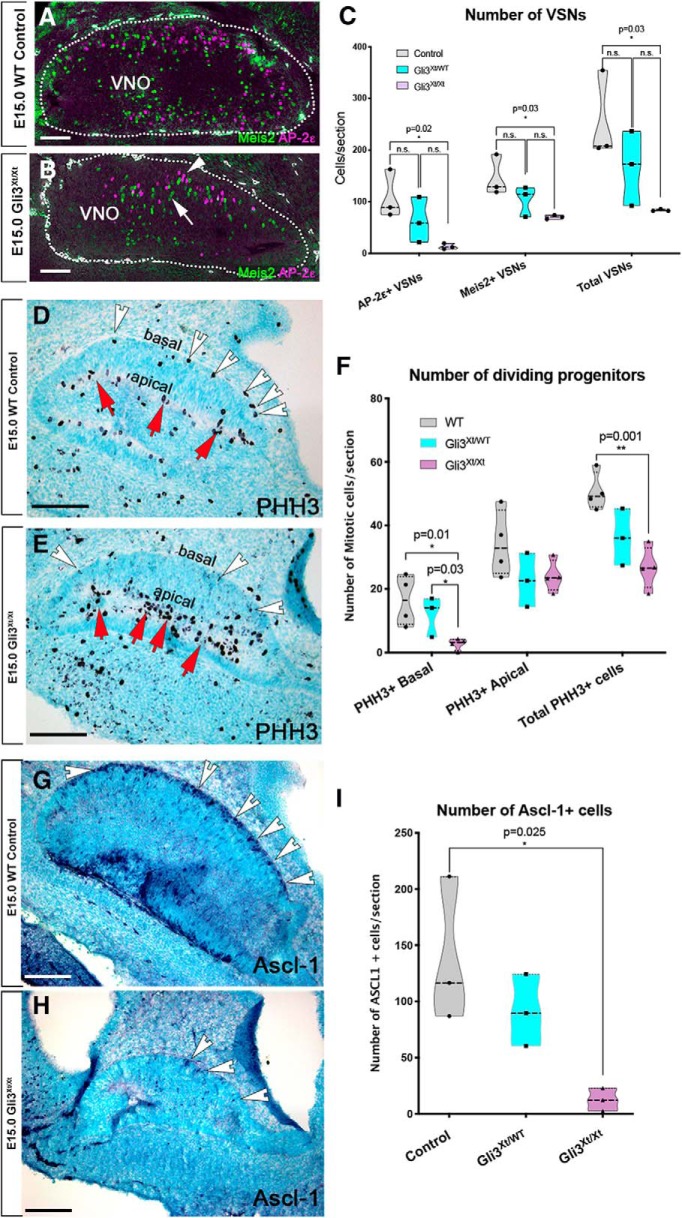
Gli3 loss-of-function impairs the formation of Ascl-1^+^ neuronal progenitor cells and VSN neurogenesis but not VSN terminal differentiation. ***A***, ***B***, Immunostaining against the transcription factors Meis2 and AP-2ε highlights maturing apical and basal vomeronasal sensory neurons in controls (***A***) and Gli3^Xt/Xt^ mutants (***B***). ***C***, Quantifications of the average number of AP-2ε differentiated VSNs and Meis2 differentiated apical VSNs reveal a significant reduction in the number of differentiated VSNs in Gli3^Xt/Xt^ mutants. ***D***, ***E***, PHH3 immunostaining and quantification (***F***) of VNO from WT and Gli3^Xt/Xt^ reveals a reduction in the total number of dividing cells with a significant reduction of basal progenitors (white arrowheads), but not apical progenitors (red arrows). ***G***, ***H***, Immunostaining against Ascl-1 in controls and Gli3^Xt/Xt^ mutants reveals (***I***) a significant reduction in the number of Ascl-1^+^ cells in the basal domains of the developing VNO. Scale bars, 100 μm.

The expression pattern of Gli3 in the Hes1^+^ progenitors in apical region of the VNO resembles its expression in the radial glia of developing brain cortex (Hasenpusch-Theil et al., 2018). Gli3 immunoreactivity in the apical Hes1^+^ proliferative region of the VNO prompted us to analyze if Gli3 repressor activity contributes to initiating the differentiation of neurogenic progenitors (Wang et al., 2011; Hasenpusch-Theil et al., 2018). We speculated that Gli3 may play a similar role as in the cortex to stimulate the onset of neurogenic progenitors responsible for the genesis of GnRH-1ns, terminal nerve cells, and VSNs. VSNs differentiate into either apical or basal VSNs (Enomoto et al., 2011; Lin et al., 2018). To identify and quantify the cell bodies of the embryonic VSNs, we performed immunolabeling against the apical VSNs' transcription factors Meis2 and the basal VSNs' transcription factor Tfap2e (AP-2ε) on Gli3^Xt/Xt^ mutants and controls (Lin et al., 2018) (Fig. 3*A*,*B*). At E15, we detected both apical and basal VSNs in the developing VNOs of controls (Fig. 3*A*). However, we found a dramatic reduction (∼70%) in the number of VSNs in Gli3^Xt/Xt-null^ mutants. (Fig. 3*C*). Heterozygous Gli3^WT/Xt^ displayed an intermediate phenotype between controls and Gli3^Xt/Xt^ mutants. These data suggest that lacking functional Gli3 compromises VSNs neurogenesis, while permitting the differentiation of the two main types of VSNs (Fig. 3*A–C*).

Quantification of PHH3^+^ mitotic cells at E15 highlighted a significant decrease in the total number of mitotic progenitors in Gli3^Xt/Xt^ mutants (Fig. 3*D–F*). However, we found a significant reduction in the number of basal progenitors in Gli3^Xt/Xt^ compared with WT animals and found no significant difference in apical progenitor cells (Fig. 3*F*). Heterozygous Gli3^WT/Xt^ embryos displayed a nonsignificant intermediate phenotype compared with WT or Gli3^Xt/Xt^. Because we found a significant reduction in proliferation in the basal portions of the VNO, we investigated whether Gli3 loss-of-function compromised the onset of Ascl-1^+^ basal neurogenic progenitors. Quantifying Ascl-1^+^ cells confirmed a dramatic (∼90%) reduction of Ascl-1^+^ neurogenic progenitors in the VNO (Fig. 3*G–I*). These data suggest that Gli3 loss-of-function in the VNO impairs the onset of neurogenic progenitors similar to what is reported for the developing brain cortex (Wang et al., 2011; Hasenpusch-Theil et al., 2018).

### Gli3 loss-of-function disrupts GnRH-1 neuronal migration

**Figure 4.**
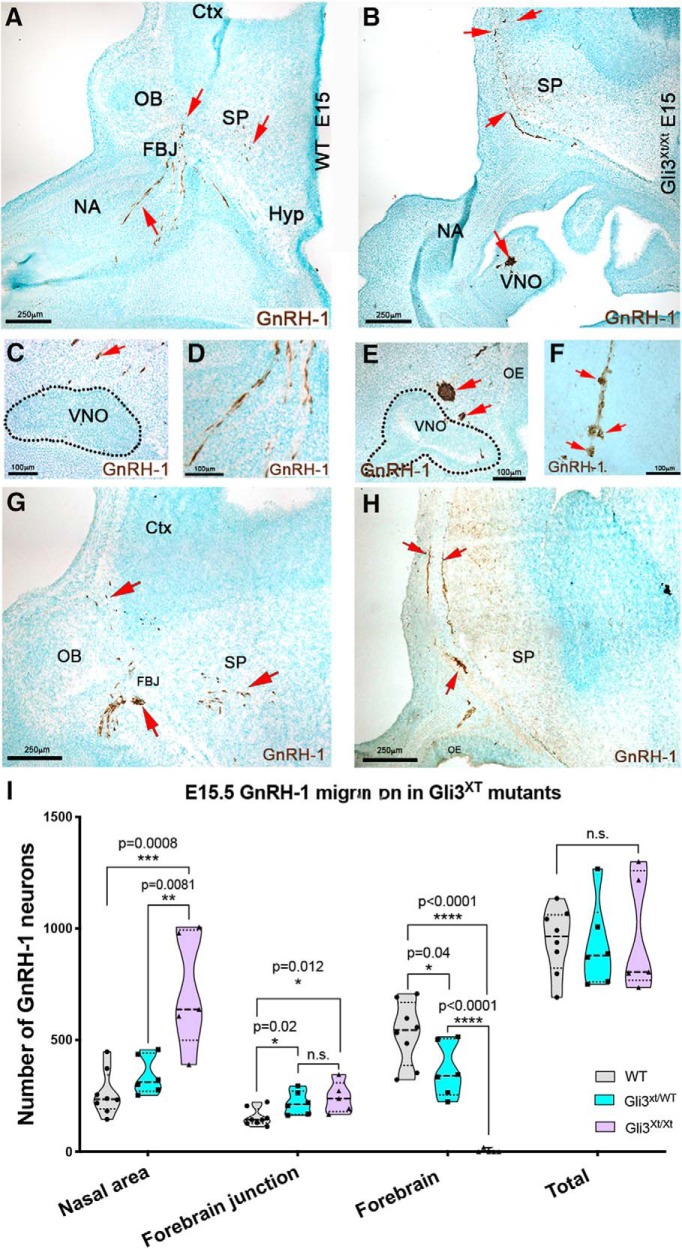
Gli3^Xt/Xt^ mutants display severely impaired GnRH-1 neuronal migration. Representative images of immunostaining against GnRH-1 on WT (***A***–***G***) and Gli3^Xt/Xt^ (***B***–***H***) at E15.5. ***A***–***G***, in WT GnRH-1ns migrate as a continuum from the vomeronasal organ (VNO) to the brain. GnRH-1ns from the nasal area (NA), cross the forebrain junction (FBJ), invade the brain ventral to the OB, migrate through the subpallium (SP) to eventually reach the hypothalamic area (Hyp). ***B***, ***C***, GnRH-1ns migrating in the nasal area and out of the VNO. ***D***, Detail showing GnRH-1ns invading the subpallium (SP) ventral to the OB in the region corresponding to the putative accumbens (Ac), some GnRH-1ns migrate to the cortex (Ctx) and around the olfactory bulbs (OBs). E-H) In Gli3^Xt/Xt^, a large portion of GnRH-1ns neurons form clusters of cells proximal to the VNO (***E***) while other migrate around the brain, occasional GnRH-1ns were found accessing the brain dorsal portion of the cortex (Ctx) (***H***). ***F***, GnRH-1 immunostaining (brown) show less organized GnRH-1ns forming clumps while migrating toward the brain (cf. ***D***). ***H***, Detail showing GnRH-1ns were unable to invade the brain migrating along the meninges. ***I***, Quantification of the distribution of GnRH-1ns in WT, Gli3^Xt/WT^ and Gli3^Xt/Xt^. In Gli3^Xt/Xt^ the majority of the GnRH-1ns remain in the nasal area. Both Gli3^Xt/WT^ and Gli3^Xt/Xt^ have a significantly smaller number of GnRH-1ns in the brain compared with controls. Values are ±SE. Dots indicate numbers of embryos/genotype, unpaired *t* test, significant values *p* < 0.05.

The observed defects in VSN development prompted us to investigate the impact of Gli3 loss-of-function on GnRH-1 neurogenesis and migration. In control conditions, GnRH-1ns migrate along the axons of the terminal nerve that invade the brain ventral to the olfactory bulb, cross the developing ventral telencephalon/subpallium, then project to the preoptic area (Cariboni et al., 2011; Hanchate et al., 2012; Giacobini and Prevot, 2013; Taroc et al., 2017) (Fig. 4*A*,*C*,*D*,*G*). However, Gli3^Xt/Xt^ mutants show large clusters of cells in the nasal area (Fig. 4*B*,*E*), as most GnRH-1ns were unable to reach the brain. Some GnRH-1ns in these mutants migrated toward the developing brain but then formed cell clumps along the migratory track (Fig. 4*F*). Sparse GnRH-1ns did reach the putative forebrain junction without invading the brain (Fig. 4*B*,*H*).

We then quantified the distribution of GnRH-1ns between the nasal area forebrain junction and brain on serial parasagittal sections after immunostaining against GnRH-1 (Fig. 4*I*). These data revealed a lack of GnRH-1-immunoreactive cells in the brain of Gli3 mutants with a massive accumulation of GnRH-1 cells in the nasal area. We observed a small, but significant, reduction in the number of GnRH-1^+^ cells in the brain of heterozygous controls at E15 with a comparable increase in GnRH-1 cells in the nasal area. Notably, quantifying WT, Gli3^Xt/WT^, and Gli3^Xt/Xt^ mutants at E15 revealed that the total number of GnRH-1ns was comparable across genotypes (Fig. 4*I*). These data indicate a distinct lineage for VSNs and GnRH-1ns. We propose that Gli3 loss-of-function compromises the formation of Ascl-1^+^ vomeronasal progenitors (Fig. 3), but not GnRH-1 neurogenesis or differentiation.

### Gli3 loss-of-function impairs OECs development in the nasal mucosa

**Figure 5.**
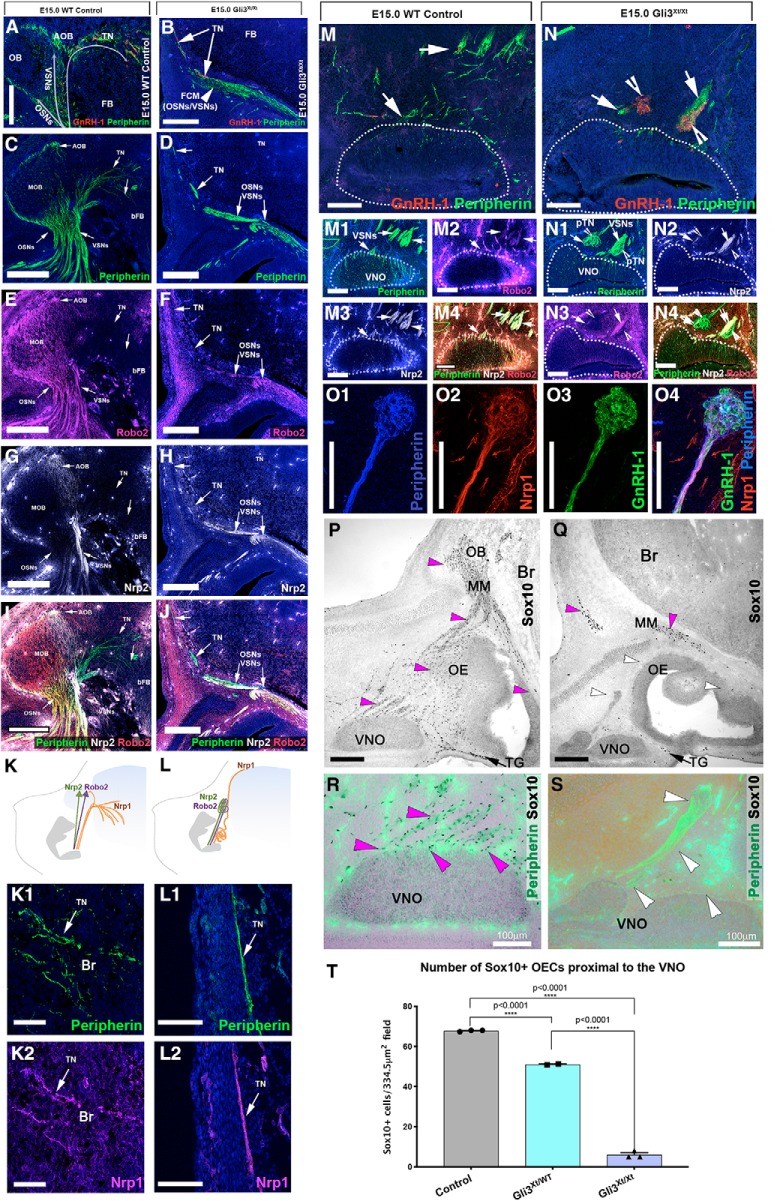
Misrouted vomeronasal and terminal nerve axons in Gli3^Xt/Xt^ are associated with lack of ensheathing cells in the nasal mucosa. ***A***, ***C***, Immunostainings against peripherin and GnRH-1. ***B***, ***D***, peripherin and GnRH-1 immunofluorescence, in Gli3^Xt/Xt^ mutants the OSNs and VSNs form a fibrocellular cellular mass (FCM). The GnRH-1ns (***D***) and the TN do not remain in the FCM but migrate around the FB. ***C***–***I***, WT E15.5, immunostaining against peripherin, Robo2, Nrp2; the TN fibers are negative for Robo2 expression and negative for Nrp2 (see merge in ***I***). ***D***–***J***, Gli3^Xt/Xt^ E15.5 immunostaining against peripherin, Robo2, and Nrp2 and merge. ***K***, ***L***, Schematic of the phenotypes and fiber immunoreactivity in controls and Gli3^Xt/Xt^ mutants. ***K1***, ***K2***, Immunofluorescence showing Neuropilin-1 (Nrp1) immunoreactivity in TN fibers invading the brain in WT embryos. ***L1***, ***L2***, peripherin and Neruopilin1^+^ fibers of the putative TN project around the brain. ***M1***–***M4***, In control animals the GnRH-1ns migrate along peripherin^+^ bundles (***M1***), also immunoreactive for Robo2 (***M2***), Nrp2 (***M3***), Merge in ***M4***. In Gli3^Xt/Xt^, GnRH-1ns form large clumps of cells suggesting disorganized peripherin^+^ fibers. Some tangled fibers of the putative terminal nerve (***N1***) are negative for Nrp2 (***N2***) and Robo2 (***N3***). Merge in ***N4***. ***O1***–***O4***, In Gli3^Xt/Xt^, tangles of putative TN fibers are positive for peripherin (***O1***) and Nrp1 (***O2***) and are associated with clusters of GnRH-1ns (***O3***). Merge in ***O4***. ***P***–***S***, GLI3^Xt/Xt^ lack of OECs associated with the vomeronasal nerve. peripherin IF and Sox10 IHC are combined. ***P***, In controls, Sox10-immunoreactive OECs (magenta arrowheads) were found around the VNO, proximal to the basal lamina of the OE, along the peripherin^+^ vomeronasal and olfactory bundles and as part of the migratory mass (MM) in front of the brain. Sox10^+^ Schwann cells were found associated along nasal trigeminal projections (TG). ***Q***, In Gli3^Xt/Xt^ mutants, Sox10-immunoreactive OECs were neither found proximal to the basal portions of the VNO, olfactory epithelium nor along the vomeronasal and olfactory projections (white arrowheads). Notably Sox10^+^ cells were found in the migratory mass (MM). As in controls Sox10^+^ Schwann cells were found associated along nasal trigeminal projections (TG). ***R***, ***S***, Magnifications of the VNO. ***R***, In control animals Sox10^+^ OECs were found proximal to the basal lamina of the VNO, along the vomeronasal bundles. ***S***, In Gli3^Xt/Xt^ Sox10^+^ OECs were rarely found in proximity of the basal lamina of the VNO and along the vomeronasal/terminal nerve bundles (white arrowheads). ***T***, Quantification of Sox10^+^ OECs around the VNO, ****p < 0.0001. Scale bars, 100 μm.

We investigated if the defective migration of GnRH-1ns in Gli3^Xt/Xt^ results from defective vomeronasal development and/or aberrant terminal nerve projections to the brain. So, we performed a series of immunolabeling experiments to discriminate between cell types. Immunolabeling with anti-peripherin provided a shared marker to highlight olfactory, vomeronasal, and terminal nerve neurons (Fig. 5*A–D*,*M*,*N*) (Taroc et al., 2017). To follow the projections of VSNs and the terminal nerve (see diagram in Fig. 5*A–D*), we performed immunolabeling against the guidance receptors, Neuropilin-2 (Nrp2), Roundabout-2 (Robo2), and Neuropilin-1 (Nrp1). Robo2 highlights projections of the basal VSNs (Fig. 5*E*,*F*,*I*,*J*), Nrp2 highlights apical VSNs (Fig. 5*G–J*) (Prince et al., 2009), while Nrp1 (Fig. 5*K2*,*L2*) (Taroc et al., 2017) labels projections of putative terminal nerve neurons (Fig. 5*K1–L2*). In control animals, we found TN fibers (Nrp1^+^) invaded the brain, together with GnRH-1ns (Fig. 5*A*,*C*,*E*,*G*,*I*). However, Gli3^Xt/Xt^ mutants showed stalled olfactory and vomeronasal axons (positive for Robo2 and/or Nrp2, Fig. 5*B*,*D*,*F*,*H*,*J*) (Balmer and LaMantia, 2004) in front of the forebrain, which formed a fibro-cellular mass (FCM). However, we did observe that sparse putative terminal nerve axons and GnRH-1ns (Fig. 5*B*,*L1*,*L2*) did reach the putative forebrain junction and project around the dorsal portions of the brain. However, the majority of GnRH-1ns (Fig. 4*E*) and terminal nerve fibers (peripherin^+^; Nrp1^+^, Fig. 5*O1–O4*; negative for Nrp2 and Robo2, Fig. 5*N–N4*) formed large tangles in the nasal area.

GnRH-1 neuronal development strictly depends on correct formation of OECs (Barraud et al., 2013; Pingault et al., 2013). Gli3 can act as a Sox10 modifier (Matera et al., 2008). Our data show that Gli3 loss-of-function leads to defective vomeronasal and terminal nerve trajectories, which phenocopies that described after OECs loss in Sox10-null mouse mutants (Barraud et al., 2013; Pingault et al., 2013). So, we tested the role of OECs in axonal misrouting and GnRH-1 migratory defects in Gli3^Xt/Xt^ mutants. So, we performed double staining against peripherin and Sox10 (Forni et al., 2011b; Pingault et al., 2013; Rich et al., 2018). In control animals, Sox10^+^ OECs were localized proximal to the basal lamina of the vomeronasal epithelium and correlated with the vomeronasal bundles emerging from the vomeronasal epithelium (Fig. 5*P*,*R*). In the OE and olfactory fibers, we observed a similar distribution with strong Sox10 immunoreactivity close to the basal regions of the OE and along the olfactory bundles projecting to the OBs (Fig. 5*P*). In Gli3^Xt/Xt^ mutants, we found a near complete lack of Sox10 immunoreactivity around the vomeronasal epithelium and no Sox10^+^ cells associated with vomeronasal axons or terminal nerve (Fig. 5*Q*,*S*). We also found no Sox10 immunoreactivity near the basal lamina of the olfactory epithelium (Fig. 5*Q*). However, in Gli3^Xt/Xt^, we found Sox10^+^ ensheathing cells proximal to the brain as part of the FCM (Fig. 5*Q*), consistent with previous reports (Balmer and LaMantia, 2004).

To determine whether the OECs in the nasal mesenchyme were missing or if they were just failing in expressing the maturation markers Sox10 (Rich et al., 2018), we performed immunostaining against BLBP which labels mature and immature OECs even in the absence of Sox10 (Pingault et al., 2013). This immunostaining indicated almost complete absence of BLBP^+^ OECs in Gli3^Xt/Xt^ mice as observed with Sox10 (data not shown). These data suggest defective OECs specification in the nasal mucosa of Gli3^Xt/Xt^.

As we detected dramatic defects in OECs formation in the developing olfactory system, we propose that the aberrant neuronal patterning and GnRH-1 migratory impairment observed in Gli3^Xt/Xt^ mutants is secondary to the lack of OECs in the nasal mucosa (Barraud et al., 2013; Pingault et al., 2013; Rich et al., 2018).

### Delayed, but not defective, migration in Gli3^Xt/WT^ heterozygous mutants

**Figure 6.**
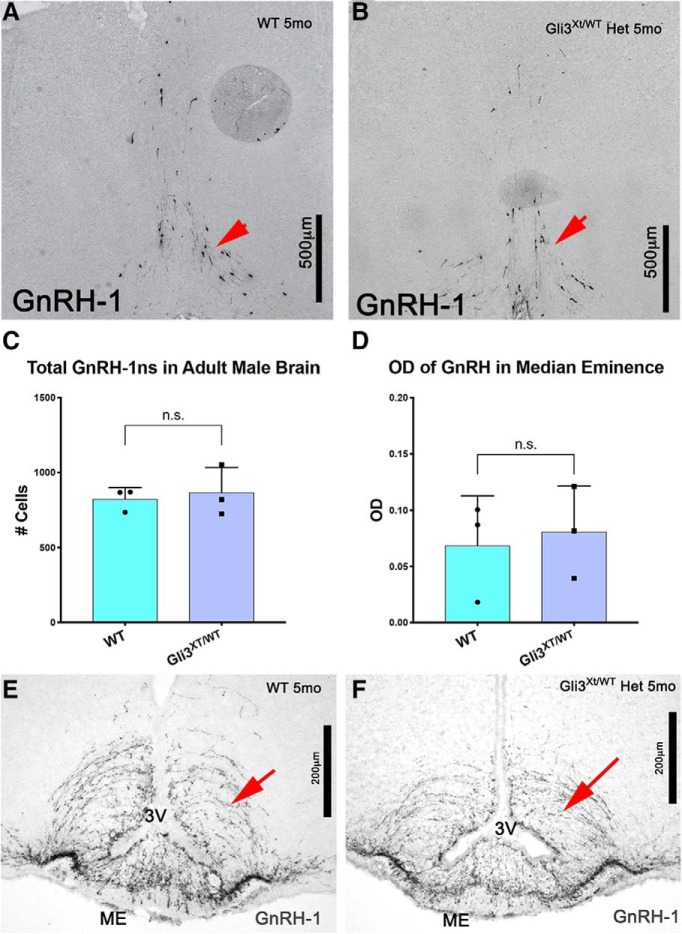
Gli3^Xt/WT^ show no difference in GnRH-1ns numbers in the brain after birth. ***A***, ***B***, Immunostaining against GnRH-1 on postnatal WT and Gli3^Xt/WT^ heterozygous animals shows comparable immunoreactivity in GnRH-1 cell bodies in the preoptic area (arrows). ***C***, Quantification for GnRH-1-immunoreactive cell bodies in the preoptic area indicates no statistical differences between controls and Gli3^Xt/WT^ heterozygous mutants. ***D***, Quantification of area occupied by GnRH-1-immunoreactive fibers in the median eminence of WT controls and Gli3^Xt/WT^ heterozygous mutants. ***E***, ***F***, Representative images showing GnRH-1 immunoreactivity of GnRH-1 axons in the median eminence (ME) of WT and Gli3^Xt/WT^ heterozygous mutants.

Partial defects in GnRH-1 neuronal migration in Gli3^Pdn/Pdn^ Gli3 hypomorphs (Naruse et al., 1994) suggest that defects in Gli3 levels can impair GnRH-1 neuronal migration. As we collected evidence indicating that Gli3 controls neurogenesis of VSNs, we further analyzed the development of the GnRH-1 system in Gli3^Xt/WT^ heterozygous embryos. Gli3^Xt/WT^ are viable and fertile, and have mild craniofacial defects and an extra toe (Veistinen et al., 2012). However, they do not display dramatic abnormalities in the olfactory system. We analyzed GnRH-1 neuronal migration in Gli3^Xt/WT^ heterozygous animals after development. Analysis of adult WT controls and Gli3^Xt/WT^ mutants (Fig. 6*A–C*) revealed no significant differences in the number and distribution of GnRH-1ns in the brain after development. Immunostaining against GnRH-1 and quantifying the average area of GnRH-1-immunoreactive axons also revealed comparable GnRH-1 innervation of the median eminence (Fig. 6*D–F*). These data suggest that Gli3 haploinsufficient mice show delayed, but not disrupted, GnRH-1 neuronal migration.

### Lack of TN and GnRH-1 invasion of the brain and altered Sema3A expression in Gli3^Xt/Xt^ mutants

**Figure 7.**
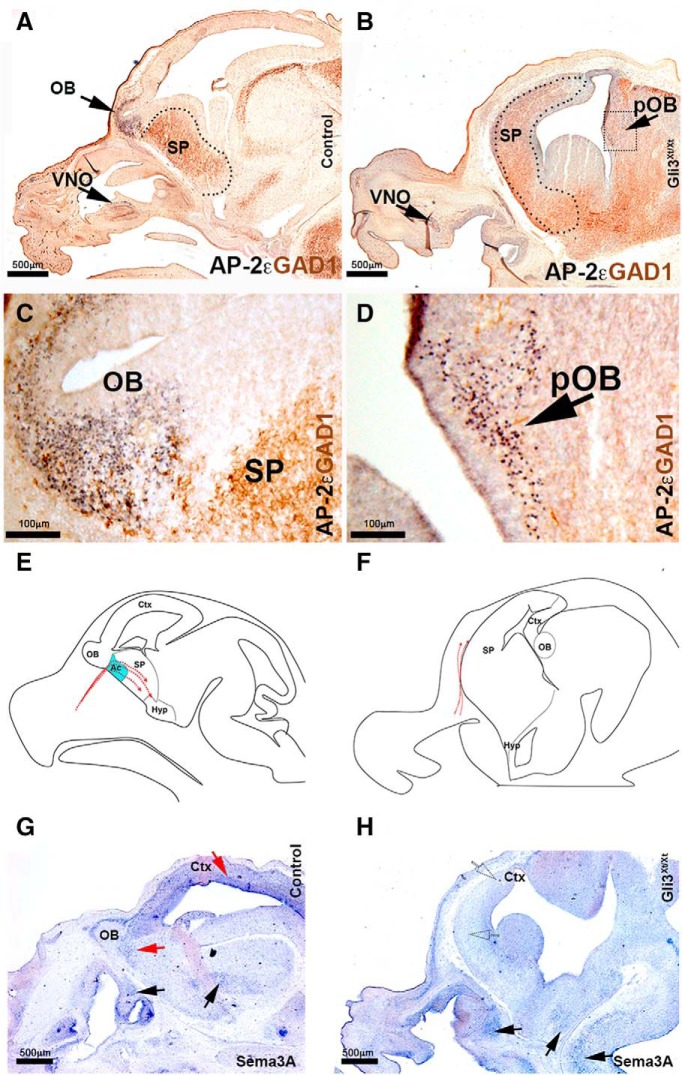
Gli3^Xt/Xt^ mutants display an expansion of expression of forebrain ventral markers, form ectopic olfactory bulbs and lack of Sema3A expression in the forebrain. ***A***, AP-2ε/GAD1 double immunostaining. Immunostaining against AP-2ε highlights mitral cells in the OB (magnified in **C**). GAD1 immunostaining highlights GABAergic neurons which are mostly located in the subpallium. ***B***, In Gli3^Xt/Xt^, AP-2ε highlight extopic mitral cells in the posterior portion of the forebrain (boxed, see magnification in (***D***) showing the ectopic AP-2ε^+^ OB in the posterior portion of the brain. GAD1 expression shows expansion of ventral forebrain maker. ***E***, ***F***, Illustration of GnRH-1ns migration in WT and Gli3-null animals. Area of Sema3A expression represented in light blue present in WT accumbens absent in the Gli3 KOs. ***G***, ***H***, *In situ* hybridization against Sema3A shows detectable Sema3A expression in the nasal area, basal brain (black arrows) and hindbrain of control and Gli3 mutants. No Sema3A expression was detected in Gli3^Xt^ mutants in the forebrain and cortex (compare red arrows in ***I*** with dotted arrows in ***H***). Ac, Accumbens; Ctx, cortex; Hyp, hypothalamus; OB, olfactory bulb; pOB, putative olfactory bulb; SP, subpallium.

In Gli3^Xt/Xt^, the few TN and GnRH-1 neurons that reach the brain fail to invade it. Because brain development can be highly dysmorphic after Gli3 loss-of-function (Aoto et al., 2002; Balmer and LaMantia, 2004; Fotaki et al., 2006; Blaess et al., 2008; Veistinen et al., 2012; Hasenpusch-Theil et al., 2018), we analyzed how such aberrant development can indirectly compromise the ability of TN and GnRH-1 neurons that do reach the brain to invade it (Figs. 4*H*; 5*K*,*L*). In rodents, gammGABA-ergic interneurons form in the ventral telencephalon and subsequently migrate toward the cortex (Anderson et al., 1997; Lavdas et al., 1999; Wichterle et al., 1999, 2001; Markram et al., 2004). The transcription factor AP-2ε can identify the developing olfactory bulbs and vomeronasal neurons (Feng et al., 2009; Enomoto et al., 2011; Lin et al., 2018) (Fig. 7*A–D*). We used antibodies against glutamate decarboxylase 1 (brain, 67 kDa) (GAD67/GAD-1) to label the ventral forebrain/subpallium. Consistent with previous reports (Yu et al., 2009; Besse et al., 2011), Gli3^Xt/Xt^ mutants exhibit a lack of rostral olfactory bulb protrusions but form ectopic olfactory bulb-like structures, positive for AP-2ε in the dorsal/lateral portions of the developing telencephalon (Fig. 7*B*,*D*). At E15.5, GnRH-1ns invade the brain by crossing the putative accumbens (ventral to the OB) and then steering ventrally toward the hypothalamic area.

During embryonic development, Sema3A is expressed ventral to the OB in the putative accumbens/ “septostriatal transition area” (Fig. 7*E*,*G*) (Taroc et al., 2017). Lack of Sema3A is not compatible with normal invasion of the brain by the TN and GnRH-1ns (Cariboni et al., 2011). As the development of the brain appears highly dysmorphic in Gli3 mutants (See diagram in 7F), we analyzed the pattern of Sema3A expression compared with controls. Our analysis revealed that Gli3^Xt/Xt^ mutants showed comparable Sema3A expression to controls in nasal areas and the hindbrain. However, we found a near complete absence of Sema3A expression in the expanded subpallium and the dorsal portion of the forebrain (Fig. 7*F*,*H*). These data show aberrant Sema3A expression and patterning in the brain of Gli3^Xt/Xt^ mutants, which, alone, is a molecular condition sufficient to prevent migratory GnRH-1 neurons to invade the brain (Cariboni et al., 2011; Taroc et al., 2017). Analysis of Sema3A expression in heterozygous Gli3^Xt/WT^ animals did not highlight obvious changes in Sema3A expression pattern compared with controls (data not shown). These data, together with the delayed but correct migration of GnRH-1ns, suggest that Gli3 haploinsufficiency is not sufficient to disrupt the patterning of the guidance cue Sema3A.

### Ascl-1 loss-of-function compromises VNO and GnRH-1 neurogenesis but retains OECs formation in the nasal mucosa

The negative effects of Gli3 loss-of-function on VSNs' but not on GnRH-1ns' neurogenesis prompted us to test 1) if Ascl-1 controls GnRH-1 neurogenesis (Kramer and Wray, 2000; McNay et al., 2006) and 2) if the lack of OECs in the nasal mucosa and impaired GnRH-1 migration, observed in Gli3 mutants, directly result from the compromised vomeronasal neurogenesis (Cau et al., 1997, 2002). For this purpose, we analyzed GnRH-1, VSNs, and OECs development on Ascl-1 KOs and controls at E13.5.

**Figure 8.**
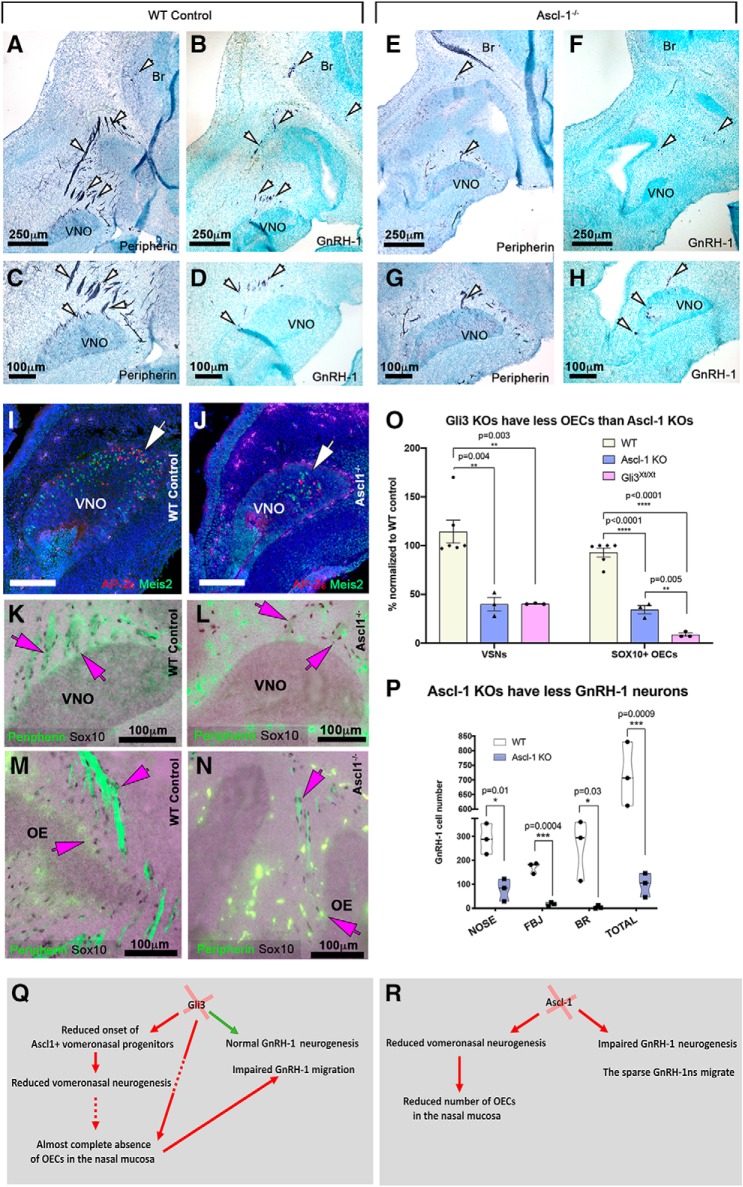
Ascl-1 KO have a comparable reduction in the number of vomeronasal and GnRH-1 neurons. ***A***–***C***, Immunostaining against peripherin highlights neuronal projections (white arrows) emerging from the VNO of WT animals projecting toward the brain. ***B***, ***D***, In controls, immunostaining against GnRH-1 reveals migratory GnRH-1ns distributed between the VNO and the brain. ***E***, ***G***, In Ascl-1-null mutants, only sparse peripherin^+^ projections emerge from the OE and VNO (white arrows). ***F***–***H***, In Ascl-1 KOs, GnRH-1ns emerge from the VNO migrating in the nasal area (white arrows). ***I***, ***J***, AP-2ε and Meis2 immunostaining highlights the reduced number of differentiated VSNs in Ascl-1 KOs (white arrow). ***K***–***N***, Reduced olfactory and vomeronasal neurons in Ascl-1 KOs does not prevent ensheathing cell formation in the nasal mucosa. IF anti peripherin and Sox10 IHC are combined. ***K***, ***M***, In control animals, many Sox10-immunoreactive OECs (magenta arrows) are found around the VNO, along the peripherin^+^ vomeronasal bundles, (***M***) proximal to the developing OE and along the olfactory bundles. ***L***, In Ascl-1^−/−^ mutants, Sox10-immunoreactive OECs were found associated with the sparse vomeronasal projections (magenta arrows) (***N***) proximal to the developing OE and along the olfactory bundles (magenta arrows). ***O***, Quantification of GnRH-1ns in controls and Ascl-1 KOs shows dramatic reduction in the number of GnRH-1ns in Ascl-1 KOs. Forebrain junction (FBJ). Holm-Sidak's multiple-comparisons test. ***P***, Quantification of VSNs, OECs, show similar defects in vomeronasal neurogenesis between Ascl-1^−/−^ and Gli3^Xt/Xt^ compared with WT controls however, more severe loss of OECs was found in Gli3^Xt/Xt^ compared with Ascl-1^−/−^. White scale bars are 100 μm. ***Q***, ***R***, Diagrams summarizing key phenotypes observed in (***Q***) Gli3^Xt/Xt^ and (***R***) Ascl-1^null^ lines.

In Ascl-1 KOs we found a (∼60%) reduction in VSNs compared with WT controls which is similar that observed in Gli3^Xt/Xt^ (Fig. 8*I*,*J*,*O*). As for the reduced neurogenesis, in Ascl-1^null^ animals we observed a comparable (−60%; SE ± 1%; *p* = 0.003) reduction in the number of OECS (Fig. 8*K–N*,*O*). However, though reduced in number, SOX10^+^ OECs were found to be distributed with similar frequency to the controls along the few vomeronasal projections (WT 0.080 cell/μm *n* = 3; Ascl-1 KO = 0.082 cell/μm, *n* = 3; *p* = 0.89).

In Ascl-1 KOs, we found sparse GnRH-1-immunoreactive neurons emerging from the developing VNO and migrating toward the brain along peripherin^+^ fibers (Fig. 8*A–H*). However, cell quantifications revealed a dramatic (∼86%) decrease in the total number of GnRH-1-immunoreactive neurons in Ascl-1 KOs (Fig. 8*P*). Analysis of Ascl-1^+/−^ revealed no differences compared with WT controls, suggesting no dose-dependent effect of Ascl-1 on vomeronasal and GnRH-1 neurogenesis (data not shown).

As we observed a severe reduction in the total number of GnRH-1ns in Ascl-1 null, but not in Gli3^Xt/Xt^, mutants, our data suggest that the mechanisms controlling Ascl-1 expression in GnRH-1 neuronal progenitors are independent of Gli3 control. Further, these observations suggest that the near complete absence of Sox10^+^ OECs in the nasal mucosa of Gli3^Xt/Xt^ mutants is not a only the result of defective vomeronasal neurogenesis (Fig. 8*O*) (Miller et al., 2018; Rich et al., 2018), but likely secondary to neural crest defects (Matera et al., 2008). In support of this conjecture, we observed that Gli3^Xt/WT^/Ascl-1^+/−^ double mutants were indistinguishable from Gli3^Xt/WT^ single mutants (data not shown).

### *A GLI3* loss-of-function mutation in a KS and polydactyl patient

**Figure 9.**
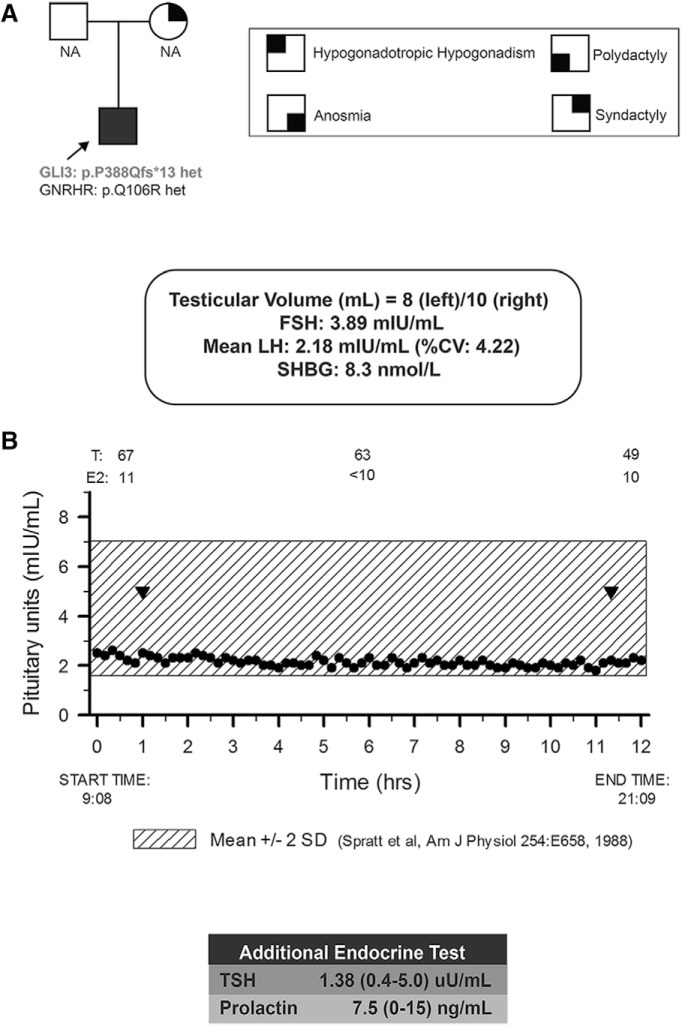
***A***, Pedigree of Kallmann syndrome proband (shown with arrow) with reproductive and nonreproductive phenotypes. ***B***, KS proband underwent neuroendocrine profiling (Q 10 min × 12 h) to chart GnRH-induced LH secretion showing low amplitude, low-frequency pulses (inverted triangles indicate LH pulses). Shaded region represents the normal reference range. NA, Not available; T, total testosterone; LH, luteinizing hormone; FSH, follicle stimulating hormone; SHBG, sex-hormone binding globulin' TSH, thyroid-stimulating hormone.

Because KS features reproductive failure and various defects in GnRH-1 neuronal migration, we first examined the genetic constraint for *GLI3* gene in Genome Aggregation Database (gnomAD) v.2.1 (Karczewski et al., 2019). This population genetics data showed that *GLI3* human genetic variants were highly constrained for loss-of-function variants with a *pLI* of 1.0 and an observed/expected (*oe*) metric of 0.09 (upper bound *oe* confidence interval of 0.20). The suggested threshold for mendelian case analysis is an upper bound *oe* confidence interval of <0.35. These parameters suggest an extreme intolerance to loss-of-function variants reflecting the strength of selection acting on heterozygotes. So, we queried WES data from 632 KS probands for *GLI3* loss-of-function variants. We identified two qualifying *GLI3* loss-of-function variants from WES data. Sanger sequencing confirmed one novel heterozygous *GLI3* loss-of-function (frameshifting indel) variant (c.1161del; p.P388Qfs*13; minor allele frequency in gnomAD: 0) in a KS male subject. This frameshift (fs) mutation leads to the formation of a premature TGA stop codon in position 1199–1201. Because parental DNA samples were not available, the precise mode of inheritance of the variant could not be evaluated. Additional KS/nIHH gene analysis showed that this KS proband also harbored a heterozygous *GNRHR* missense mutation (p.Q106R) (Fig. 9). Because gnomAD variants are not Sanger-confirmed, we performed a gene-based burden test between the KS cohort and gnomAD for all qualifying *GLI3* loss-of-function variants that showed enrichment in the KS cohort (*p* = 0.002). We did not observe this enrichment when correcting for Sanger-confirmed variants only in the KS cohort but retaining all unconfirmed gnomAD variants (*p* = 0.06).

The patient harboring the *GLI3* loss-of-function variant is a 38-year-old male, who presented to medical attention at age 18 years with anosmia and failure to enter puberty. He was undervirilized with a bilateral low testicular volume (Fig. 9). His biochemical investigations showed frank hypogonadotropic hypogonadism (serum testosterone: 67 ng/dL; LH: 2.18 mIU/ml and FSH: 3.89 mIU/ml) with neuroendocrine studies revealing low-amplitude low-frequency LH pulse profile consistent with hypogonadotropism (Fig. 9). The remaining pituitary hormone profile was normal and UPSIT showed complete anosmia. These results confirmed a clinical diagnosis of Kallmann syndrome. Consistent with the well established role of GLI3 in limb development, he reported in his history an extra toe on his right foot, for which he underwent surgery as a child, and bilateral webbed toes (second and third toes). Although the precise inheritance mode of the variant was not determined due to lack of parental samples, his mother also exhibited syndactyly affecting her feet, suggesting a possible autosomal-dominant mode of inheritance with variable penetrance for the reproductive phenotype (Fig. 9).

**Figure 10.**
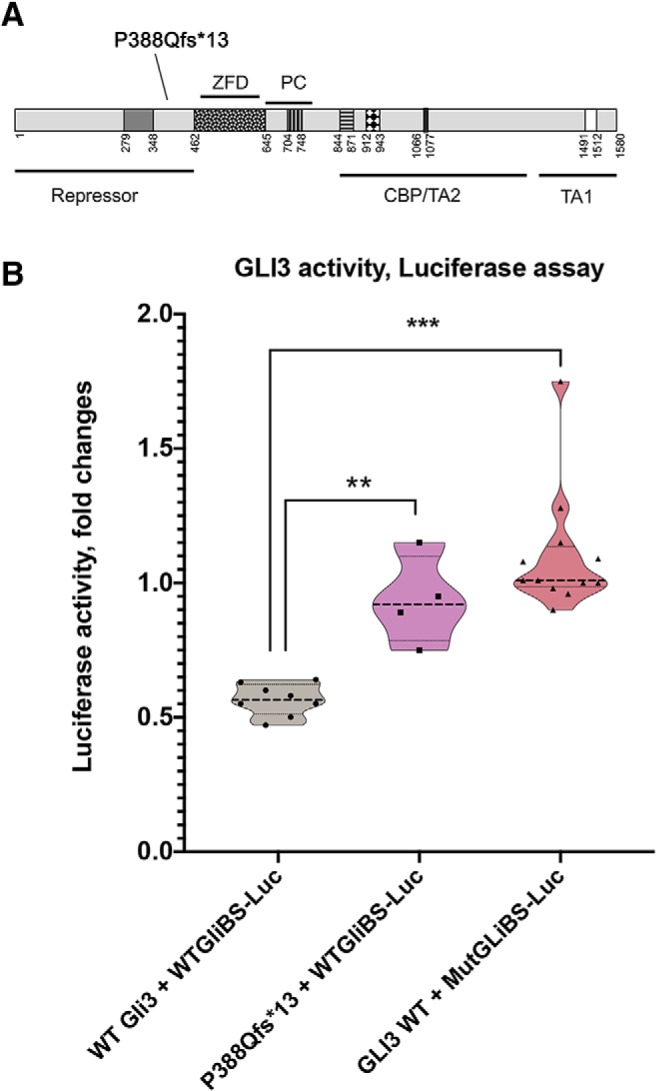
Functional validation of GLI3 loss-of-function. ***A***, Diagram illustrates *GLI3* protein, the mutation P388Qfs*13 in the repressor domain of the protein. ZFD, Zinc finger domain; PC, protein cleavage; TA1, TA2, CBP-binding domain, transactivation domain 1 and 2. ***B***, Luciferase activity assay, the repressor activity of Gli3WT on the reporter (WTGLiBS-Luc) is lost in the truncated GLI3. No repression was found for WT Gli3 on reporter carrying nonfunctional Gli binding site MutGLiBS-Luc. **p = 0.002, ***p < 0.0001, Holm–Sidak's multiple-comparisons test.

To test the biological effects of the identified GLI3 variant, we performed a luciferase assay (Fig. 10) to compare the activity of Wild-type (WT) GLI3 and mutated GLI3 using a reporter plasmid containing wild-type GLI binding site linked to firefly luciferase (WTGliBS-Luc). A reporter carrying a mutated/nonfunctional (MutGLiBS-Luc) Gli3 binding site linked to firefly luciferase (Sasaki et al., 1997; Saitsu et al., 2005) served as negative control. In contrast with a prior report (Krauss et al., 2008), luciferase assay on HeLa cells (ATCC CCL-2) revealed that Gli3 acts a transcriptional repressor. This analysis demonstrated the deleterious loss-of-function nature of our novel *GLI3* frameshift mutation (p.P388Qfs*13), which affected the repressor ability of Gli3, consistent with a GLI3 loss-of-function as a putative mechanism for GLI3-related KS.

## Discussion

Here, we found that Gli3 loss-of-function induces: (1) loss of Ascl-1 neuronal progenitors in the VNO and dramatically reduces the number of vomeronasal sensory neurons (VSNs), (2) normal neurogenesis for GnRH-1ns, 3() nearly complete absence of GnRH-1 neuronal migration, associated to aberrant formation of OECs in the nasal mucosa, and (4) loss of forebrain expression of Semaphorin 3A (Sema3A), a key axonal guidance molecule that controls GnRH-1 invasion of the brain (Cariboni et al., 2011; Hanchate et al., 2012; Young et al., 2012). To elucidate the underlying cellular substrates of the observed phenotypes, we analyzed Ascl-1^−/−^ mutants. Even though we found reduced vomeronasal neurogenesis similar to Gli3 mutants, Ascl-1^−/−^ mutants showed a severely reduced number of GnRH-1ns' and Sox10^+^ OECs (OECs) that formed along axonal bundles similar to controls (Fig. 8). Based on the observed phenotypes between Gli3 and Ascl-1 mutants, we conclude that Ascl-1 is crucial for GnRH-1 neurogenesis independent of Gli3 control. Ascl-1^−/−^ mice showed that reduced neurogenesis in the VNO alone does not prevent the maturation of Sox10^+^ OECs in the nasal mucosa and the migration of GnRH-1ns in Gli3 mutants. These data suggest a direct role for Gli3 in controlling OECs development. These murine observations highlight a critical role for Gli3 distinctly in OEC development and GnRH neuronal migration, two features that underlie the pathogenesis of KS. In keeping with this notion, we also identified and functionally validated a novel GLI3 loss-of-function mutation in a KS patient who also exhibited polydactyly, a well recognized phenotypic feature of GLI3-related human diseases.

Analyzing Gli3 expression in the developing olfactory pit, we observed that, Gli3 is expressed by proliferative progenitors in the apical portions of the developing VNO positive for the transcription factor Hes-1 (Figs. 1,2). Hes1 inhibits neurogenesis by repressing the transcription factor Ascl-1 and maintaining neuroepithelial cells in undifferentiated/proliferative state (Ohtsuka et al., 1999; Cau et al., 2000; Ohtsuka et al., 2001). Neurogenic Ascl-1 progenitors were found to be distributed in the basal portions of the developing VNO (Fig. 2). In Gli3-null mutants we observed a dramatic reduction of VSNs secondary to a near complete loss (∼91%) of Ascl-1^+^ neurogenic progenitors in the VNO. Surprisingly, the number of GnRH-1ns did not appear different from WT controls. These results indicate that loss of Gli3-mediated gene repression negatively regulates the onset of vomeronasal neurogenic progenitors, but not GnRH-1 neurogenesis.

The cyclin-dependent kinase gene Cdk6 was recently identified as a direct target of Gli3-mediated repression (Hasenpusch-Theil et al., 2018). Gli3 loss-of-function shortens the cell cycle and reduces neurogenesis (Pilaz et al., 2009; Hasenpusch-Theil et al., 2018). Because we detected Gli3 in Hes-1 proliferative cells, we hypothesize that Gli3 controls the onset of neurogenesis through a similar mechanism as in the cortex (Hasenpusch-Theil et al., 2018). Active Shh signaling induces Gli1 expression. By using a Gli1-Lac-Z mice, we observed lack of active Shh signaling in the VNO. This finding suggests, together with the absence of GnRH-1 phenotypes in Gli1/Gli2 KOs (Metz and Wray, 2010), that Gli3 acts as a transcriptional repressor in the developing VNO.

Gli3 heterozygous mice showed a delay in GnRH-1 migration without differences in cell number or distribution after birth and were fertile. These data indicate that heterozygous Gli3 loss-of-function mutations, alone, do not produce a GnRH-deficient state in mice, which is consistent with results from Gli3 hypomorphic mutant mice (Naruse et al., 1994).

OECs are a glial population that wraps bundles of olfactory and vomeronasal bundles (Rich et al., 2018). OECs promote normal GnRH-1 neuronal migration from the nasal area to the brain (Barraud et al., 2013; Geller et al., 2013; Pingault et al., 2013). OECs arise from the neural crest precursors (Barraud et al., 2010; Forni et al., 2011b; Miller et al., 2016, 2018; Rich et al., 2018). By analyzing GnRH-1ns migration in Gli3^Xt/Xt^ mutants, we found that most GnRH-1 cells were unable to migrate far from the VNO and formed large clumps of cells on tangled fibers of the putative TN. This phenotype matches that of Sox10-null mutants (Barraud et al., 2013; Pingault et al., 2013). Gli3 can act as a Sox10 modifier, which is consistent with the suggestion that Gli3 loss-of-function can compromise OECs development (Matera et al., 2008). Here, we found virtually no Sox10 or BLBP^+^ cells in the olfactory/vomeronasal bundles of Gli3^Xt/Xt^ mutants nor proximal to the basal lamina and lamina propria of the olfactory epithelia, suggestive of Gli3 loss impairing OECs development in the nasal mucosa. In Gli3^Xt/Xt^ mice, we found Sox10^+^ Schwann cells around the trigeminal nerve (Fig. 5) and around the fibers of the sensory Grueneberg ganglion (data not shown) (Liu et al., 2009; Matsuo et al., 2012; Moine et al., 2018). We also found Sox10^+^ OECs surrounding the FCM proximal to the OBs. These intriguing data suggest developmental differences between OECs of the nasal mucosa and olfactory bulb OECs (Richter et al., 2005; Ito et al., 2006; Mayeur et al., 2013).

In Gli3^Xt/Xt^ mutants, we found sporadic TN fibers and GnRH-1 cells could reach the brain but not invade it. These findings revealed a similar cellular behavior to that described after Sema3A loss-of-function. Analyses of Gli3^Xt/Xt^ mutants confirmed very dysmorphic forebrain structures with expanded regions positive for the early subpallial marker, GAD1, and ectopic OBs (Balmer and LaMantia, 2004; Fotaki et al., 2006; Blaess et al., 2008; Veistinen et al., 2012). *In situ* hybridization against Sema3A showed a near complete absence of Sema3A expression in the forebrain, a condition that alone is not permissive for normal GnRH-1 migration (Cariboni et al., 2011). We propose that altered Sema3A expression in Gli3 mutants directly reflects either the lack of functional Gli3 transcriptional control or overall aberrant brain development.

Analyzing Ascl-1^null^ mutants revealed a role for Ascl-1 in controlling GnRH-1 neuronal development, since we found an approximate 86% reduction in the number of GnRH-1ns in these mice.

We also found no vomeronasal defects together with normal GnRH-1 cell numbers and migration in Ascl-1^+/−^ heterozygous mice. This finding indicates that Ascl-1 is not required in a dose-dependent manner for vomeronasal and GnRH-1 neurogenesis as described for other neurons (McNay et al., 2006). The lack of overlapping phenotype between Gli3^Xt/Xt^ (normal GnRH-1 but reduced VSN cell numbers) and Ascl-1^null^ mutants (reduced GnRH-1 and VSNs cell numbers) indicates that GnRH-1 neurogenic onset is not regulated in a Gli3-dependent fashion.

We observed that OECs defects in Ascl-1^null^ mutants appeared less severe than in Gli3^Xt/Xt^ and that OECs in the mucosa of Ascl-1^null^ mutants were positive for Sox10 (Fig. 8*O*). Moreover, Gli3^Xt/WT^/Ascl-1^+/−^ double mutants did not have exacerbated phenotypes compared with Gli3^Xt/WT^ suggesting that the effects seen in both Ascl-1^null^ and Gli3^Xt/Xt^ are independent of each other and not additive (data not shown). We conclude that the near complete absence of OECs in the nasal mucosa of Gli3^Xt/Xt^ mutants is not secondary to the decreased number of vomeronasal fibers (Miller et al., 2018).

Analysis of WES from KS/nIHH patients identified a novel frameshift (fs) *GLI3* variant (p.P388Qfs*13) that causes the formation of a premature TGA termination codon in position 1199–1201. Using site-directed mutagenesis and luciferase assays, we confirmed a loss of repressor ability of this mutation, which decreased the repressor ability of GLI3. This patient also displayed polydactyly and syndactyly, two hallmark clinical features of humans with *GLI3*-Greigs cephalopolysyndactyly syndrome (GCPS), which corresponds to the loss of GLI3 transcriptional repression during limb development (Johnston et al., 2010; Al-Qattan et al., 2017). This mutation affects the 5′ part of the *GLI3* gene upstream of the zinc finger domain which is well correlated with GCPS phenotype (Johnston et al., 2010; Al-Qattan et al., 2017). Reviewing previously reported *GLI3* mutation subjects with GCPS or oral-facial-digital syndrome indicates that a small minority of these patients also display micropenis (Kroisel et al., 2001; Johnston et al., 2010), cryptorchidism (Kroisel et al., 2001; Johnston et al., 2010), or anosmia (Johnston et al., 2010). These phenotypes are consistent with an underlying KS. KS/nIHH may be under-recognized in these reports, as reproductive phenotypes cannot be assessed in prepubertal subjects harboring *GLI3* mutations. Our findings implicate heterozygous *GLI3* loss-of-function mutations as a contributing genetic factor for KS/nIHH, which is further supported by the associated *GLI3* missense variants in two subjects with HH (Quaynor et al., 2016).

We acknowledge that heterozygous *GLI3* mutations alone may be insufficient to cause KS/nIHH and may require additional genetic or nongenetic modifiers. Indeed, KS has a well established oligogenic basis (Sykiotis et al., 2010). Here, we found that the proband with *GLI3* loss-of-function mutation also harbored an additional rare *GNRHR* p.Q106R missense mutation. Although the *GNRHR* p.Q106R variant can be deleterious in cellular assays (Leaños-Miranda et al., 2005), *GNRHR* mutations typically cause autosomal recessive hypogonadotropic hypogonadism (Gianetti et al., 2012). So, the cooccurrence of *GLI3* and *GNRHR* variants in this proband suggests a potential oligogenic (*GLI3/GNRHR*) contribution to KS.

In summary, we revealed a crucial role for Gli3 in controlling vomeronasal sensory neurons neurogenesis, formation of OECs in the nasal mucosa, and GnRH-1 neuron migration. Our findings suggest a role for Gli3 in triggering the transition from a proliferative to neurogenic program in the developing VNO. Because we found a dramatic reduction in the number of Sox10 expressing OECs and aberrant Sema3A in the brain, we propose that Gli3 acts as a genetic modifier and contributes to the oligogenic nature of KS/nIHH. Future studies will delineate the gene regulatory network controlled by the Gli3 transcriptional network in the developing nasal olfactory system and identify KS/nIHH candidate genes to confirm the role of this transcription factor as a modifier in the oligogenic nature of KS/nIHH.
